# Viral-toxin interactions and Parkinson’s disease: poly(I:C) priming enhanced the neurodegenerative effects of paraquat

**DOI:** 10.1186/1742-2094-9-86

**Published:** 2012-05-04

**Authors:** Jessica Bobyn, Emily N Mangano, Anusha Gandhi, Eric Nelson, Kerry Moloney, Melanie Clarke, Shawn Hayley

**Affiliations:** 1 Department of Neuroscience, Carleton University, 1125 Colonel By Drive, Ottawa, ON, K1S 5B6, Canada

**Keywords:** Neuroinflammation, Neurodegeneration, Microglia, Cytokine, Pesticide, Viral

## Abstract

****Background**:**

Parkinson’s disease (PD) has been linked with exposure to a variety of environmental and immunological insults (for example, infectious pathogens) in which inflammatory and oxidative processes seem to be involved. In particular, epidemiological studies have found that pesticide exposure and infections may be linked with the incidence of PD. The present study sought to determine whether exposure to a viral mimic prior to exposure to pesticides would exacerbate PD-like pathology.

****Methods**:**

Mice received a supra-nigral infusion of 5 μg of the double-stranded RNA viral analog, polyinosinic: polycytidylic acid (poly(I:C)), followed 2, 7 or 14 days later by administration of the pesticide, paraquat (nine 10 mg/kg injections over three weeks).

****Results**:**

As hypothesized, poly(I:C) pre-treatment enhanced dopamine (DA) neuron loss in the substantia nigra pars compacta elicited by subsequent paraquat treatment. The augmented neuronal loss was accompanied by robust signs of microglial activation, and by increased expression of the catalytic subunit (gp91) of the NADPH oxidase oxidative stress enzyme. However, the paraquat and poly(I:C) treatments did not appreciably affect home-cage activity, striatal DA terminals, or subventricular neurogenesis.

****Conclusions**:**

These findings suggest that viral agents can sensitize microglial-dependent inflammatory responses, thereby rendering nigral DA neurons vulnerable to further environmental toxin exposure.

## **Background**

The neurodegenerative process that occurs in Parkinson’s disease (PD) appears to be complex, involving mitochondrial and ubiquitin-processing defects, along with alterations of oxidative, apoptotic, and trophic factors [1-5]. One common mechanism that could link virtually all pro-death pathways in PD is the microglial-dependent neuroinflammatory processes that are typically induced in the substania nigra pars compacta (SNc) of patients with PD, and in numerous animal models of the disease [6-8]. Moreover, increasing evidence supports the notion that cumulative multiple environmental toxin ‘hits’, (for example, pesticides, heavy metals, and infectious pathogens) over time might act as triggers for PD through their effects upon neuroinflammatory cascades [9,10], possibly in conjunction with genetic vulnerabilities.

It is possible that exposure to one environmental toxin ‘hit’ sensitizes neuroinflammatory or pro-death pathways, rendering nigrostriatal dopamine (DA) neurons more vulnerable to various secondary insults [11-13]. In fact, it has been shown that prenatal exposure to the bacterial endotoxin known as lipopolysaccharide (LPS) enhanced the vulnerability of SNc DA neurons to later pesticide exposure in adulthood [14]. Our own work similarly showed an augmented loss of DA neurons and protracted microglial activation in adult mice primed with a single low dose of LPS followed by later treatment with the pesticide paraquat [12]. Whatever the case, immunocompetent brain microglia are crucially involved in responding to all types of foreign insults and are likely key players in modulating neurodegenerative pathways.

Microglia possess Toll- like receptors (TLRs) which recognize pathogen-associated molecular patterns (PAMPs) found on microbes [15,16]. TLRs are ubiquitously expressed in peripheral leukocytes and microglia, and to a lesser extent astrocytes, oligodendrocytes and possibly even neurons [15]. TLR4 typically recognizes pathogens of a bacterial nature, whereas the TLR3 pathway is engaged by viral pathogens or viral- like challenges, such as double-stranded RNA, and both TLR pathways promote pro-inflammatory cytokines, chemokines, and oxidative factors [15,17]. Although TLR regulation is essential for maintaining homeostasis, excessive TLR engagement has been linked to inflammatory and neurodegenerative diseases, including Alzheimer’s disease, multiple sclerosis, and ischemic stroke [17-20].

Although little is known about the interaction of viral TLR mechanisms and PD, a few studies have reported correlations between PD-like symptoms or post-encephalic parkinsonism and a variety of viral infections [21-24]. More recently, experimental evidence indicated that the H5N1 virus induced PD-like motor disturbances, increased α-synuclein phosphorylation, and resulted in a loss of SNc DA neurons in rodents [23]. Similarly, administration of the viral mimetic and TLR3 agonist, polyinosinic: polycytidylic acid (poly(I:C)), induced long-term glial activation in the SNc and the striatum [25].

In this study, we assessed whether exposure to the viral-like challenge poly(I:C),would enhance the neurodegenerative effects of later treatment with paraquat, and whether any such effects were associated with the microglial and oxidative status of the animals. Our findings do support the contention that DA neurons are especially vulnerable to environmental insults when exposure occurs in the context of an activated microglial microenvironment stemming from viral challenge. However, the SNc pathology induced by poly(I:C) and paraquat appeared to occur in the absence of permanent behavioral or striatal deficits. These data also indicate that the duration of the augmented microglial response induced by pre-treatment with a TLR3 agonist (poly(I:C)) pre-treatment followed by paraquat administration exceeded what we previously with TLR4 stimulation (using LPS) performed before the pesticide [12]. Hence, viral and bacterial insults have the potential to interact with an environmental toxin to differentially affect the microglial state and extent of SNc pathology.

## **Methods**

All experimental tests were approved by the Carleton University Committee for Animal Care and were conducted in adherence to the guidelines stipulated by the Canadian Council for the use and care of animals in research.

### **Animals**

Male C57BL/6 mice (Charles River, Laprarie, Quebec, Canada) were obtained at 10–12 weeks of age and given 1 week to acclimatize to their new conditions before experimentation. Animals were housed singly in standard polypropylene cages, and maintained on a 12 hour light/dark cycle. Mice were provided with food and water *ad libitum*, and temperature and humidity were controlled in the rooms.

### **Cannulation and infusion**

Animals underwent stereotaxic cannula implantation (described below), with the cannulae situated directly above the SNc, and were given 1 week to convalesce before experimentation. Mice were then infused with the TLR3 agonist, poly(I:C) (5 μg/2 μl) (InvivoGen, San Diego, CA, USA) or saline. After the infusions, mice (n = 8) received intraperitoneal injections with paraquat (1,1′-dimethyl-4,4′-bipyridinium dichloride; 10 mg/kg; Sigma-Aldrich, Oakville, ON, Canada,), or an equivalent volume of saline (Sigma-Aldrich, Oakville, ON, Canada), three times per week for 3 weeks, giving a total of nine injections, as previously described [26,27]. Mice began receiving the paraquat or saline injections at 2, 7, or 14 days after intra-SNc poly(I:C) or saline infusion in order to investigate whether varying exposure times differentially affect paraquat toxicity. Treatment groups are summarized in Table 1.

**Table 1:** Breakdown of groups as a function of saline, poly(I:C) and paraquat (PQ) administration

**Treatment group**	**Infusion**	**Delay, days**	**Injection**	**Time before tissue harvest, days**
**1**	Saline	2	Saline	7
**2**	Saline	2	PQ	7
**3**	Poly I:C	2	Saline	7
**4**	Poly I:C	2	PQ	7
**5**	Poly I:C	7	PQ	7
**6**	Poly I:C	14	PQ	7

### **Surgery**

Animals underwent cannula implantation surgery as previously described [27]. Briefly, mice were anesthetized with oxygen-enriched isoflurane, and placed in a stereotaxic apparatus. All mice were then implanted with indwelling cannulae directly above the SNc (bregma: anterior–posterior −3.16 mm, ± lateral 1.2 mm, ventral −4.0 mm). Animals were given 1 week to convalesce after surgery before experimentation began. Infusions were delivered through polyethylene tubing connected to a microliter syringe (Hamilton; Reno, NV, US) and syringe pump (Harvard Apparatus Pico Plus; Holliston, MA, US). The drug was dissolved in 2 μl of saline and delivered over a 5-minute period, followed by a 2-minute pause to ensure proper delivery. Infusions were conducted between 08.30 and 14.00 hours to avoid variability attributed to diurnal variations. Furthermore, animals were unrestrained during the infusion process to minimize stress.

### **Behavioral analysis**

Home-cage locomotor activity was monitored during a complete 12-h light/dark cycle using an infrared beam-break apparatus (Micromax; Accuscan Instruments, Columbus, OH, USA). Locomotor activity was measured during the third and fourth weeks of experimentation (15 and 22 days after the infusion), and 24 hours before being killed. Animals were given 1 hour to acclimatize to the room prior to assessment. Total locomotor activity was assessed by recording the number of infrared beam-breaks over a 24-hour period, as described previously [28].

Motor coordination was examined by way of a pole test, as previously described [29,30]. This test consists of a pole 500 mm pole in length and 8 mm in diameter, with a plastic ball at the top. The pole was covered with non-toxic adhesive tape to increase traction for the animals. Home-cage bedding was placed at the bottom of the pole to encourage the animals to descend. Animals were placed head upward directly below the plastic ball. Motor coordination is necessary for the mouse to rotate downward and climb to the ground. Latency to rotate 180^o^ and latency to climb down the pole was recorded, with maximum test duration set at 90 seconds. Testing was conducted under dim lighting conditions between 09.00 and 1400 hours on the day after the second infrared recording (23 days after the infusion). The animals were recorded for three trials, with a 1 minute rest period between tests.

### **Immunohistochemical analysis**

Animals were killed with an overdose of sodium pentobarbital injection on the seventh day after the final injection, and perfused with ice-cold saline followed by 4% paraformaldehyde. The whole brain was dissected out and stored in 0.1 mol/L PBS with 20% sucrose solution and 0.02% sodium azide at 4°C until further analysis.

Fixed brains were cut on a cryostat into coronal sections 20 μm thick, and the striatum and SNc secions were mounted directly onto gelatin-coated slides, then analyzed for tyrosine hydroxylase (TH) staining as a representative measure of nigrostriatal DA neurons. SNc sections were also stained for the microglial and oxidative stress markers, CD11b and gp91, respectively.

To provide an index of potential neuroplastic changes that could influence behavioral outputs, striatal sections were stained for doublecortin (DCX). Sections were incubated overnight at 4°C in mouse anti-TH (1:1000, ImmunoStar), rat anti-CD11b (1:1000, AbDSerotec), goat anti-gp91 (1:1000) or goat anti-DCX (1:1000) (both Santa Cruz Biotechnology, Santa Cruz, CA, USA). Sections were further incubated for 2 hours at room temperature with their respective biotinylated antibodies: biotin anti-mouse (1:500), biotin anti-rat (1:500),or biotin anti-goat (1:1000) (all Jackson ImmunoResearch Laboratories, Inc., West Grove, PA, USA). Primary and secondary antibodies were diluted in 0.01 mol/l PBS (pH 7.3) containing 2% BSA with 0.3% Triton X-100 and 0.01 sodium azide. Sections were further incubated in horseradish peroxidase-conjugated streptavidin tertiary antibody (1:1000 for gp91 and DCX, 1:500 for TH and CD11b; Jackson ImmunoResearch) at room temperature for 2 hours and dissolved in 0.01 mol/l PBS (pH 7.3) containing 2% BSA and 0.3% Triton X-100. Antibodies were visualized by incubation with DAB (Sigma-Aldrich) for 10 minutes on a shaker table. TH-stained SNc sections were further counterstained with cresyl violet (Sigma-Aldrich).

The microglial state of activation on CD11b-stained sections was rated using a previously described scale [12]. In brief, cells were rated on a scale of 0–3, where 0 reflects the lowest state of activation (microglia are in their ‘resting state’, identified by highly ramified, thin processes); 1 indicates an intermediate state of activation (fewer than 10 cells within the SNc are moderately activated.); 2 indicates higher activation (more than half of the visible cells were in an intermediary or active state, characterized by rounded soma and no or few processes) and 3 indicates sections showing the highest level of activation (majority of cells exhibited a very active state).

In addition, gp91 reactivity was rated on a similar scale: 0 reflected little to no gp91 reactivity; 1 indicated low to moderate gp91 immunostaining (gp91-positive microglial cells were present in relatively low numbers); 2 signified increased gp91 staining (numerous strongly reactive microglia present).

All ratings (both CD11b and gp91) were conducted twice by the same person, who was blinded to all treatments. The ratings yielded an inter-rating reliability of over 90% , and all scores per section were averaged to give an overall representation of total nigral glial cell and oxidative activity (CD11b and gp91, respectively).

### **Quantification of tyrosine hydroxylase-positive neurons**

Quantification of SNc DA-producing neurons was achieved by serial section analysis of the number of SNc TH-positive cells, at bregma levels −3.08, -3.16, -3.28, -3.40, and −3.52. Using a double-blind procedure, the total number of TH-positive neurons was counted across multiple bregma levels for each animal in each of the treatment groups. Midbrain TH-positive counts from each bregma level and animal were compared across treatment groups. The same midbrain sections were also used to evaluate the total number of TH-negative cells, which represents the number of surviving neurons. Cresyl violet-stained neurons from each bregma level and animal were quantified and compared across treatment groups.

Striatal quantification photomicrographs were obtained for each animal using the same exposure time. Image J software was used to determine the background threshold for each striatal section and the total number of white (background) and black (TH-positive) pixels. All images were converted into an eight-bit format, where the grayscale varied from 0 to 255. The area of interest was selected and the upper and lower threshold values were used across all images to separate the features of interest from the background. The upper and lower thresholds were determined using an automatic thresholding option, a modified version of the IsoData method. This algorithm divides the image into the object of interest and background by taking an initial threshold (histogram-derived), followed by the averages of the pixels at or below the threshold, and then the pixels above are computed. The averages of those two values are computed, the threshold is incremented, and the process is repeated until the threshold is larger than the composite average. The data were then presented as a histogram with the number of black (object of interest) and white (background) pixels present.

### **Statistical analysis**

Home-cage activity was assessed using a mixed repeated-measures analysis of variance (ANOVA) with paraquat and poly(I:C) treatment effects evaluated over time. All other data were analyzed by standard ANOVA, followed by Fisher’s planned comparisons (*P* < 0.05) where appropriate. Data were evaluated using StatView statistical software package (version 5.0; SAS Institute, Inc., Cary, NC, USA)

## **Results**

### **Behavioral analysis**

**Figure 1:**
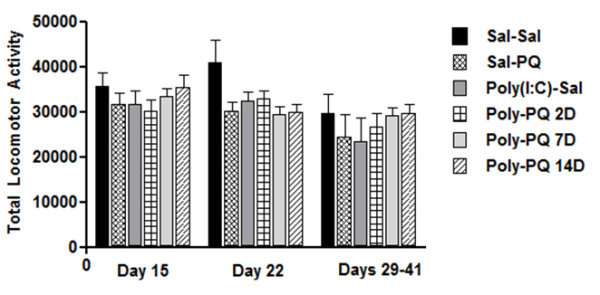
**Total home-cage locomotor activity was monitored for 24 hours at three separate time points: 15 and 22 days after poly(I:C) infusion, and 24 hours before animals were killed.** No significant differences were seen between treatment groups at any of the time intervals. However, there was a trend towards reduced activity at the middle time point (22 days). Data are expressed as a mean ± SEM; n = 8 to 15 Analysis of pole-test performance was used to investigate any PD-like hindlimb and forelimb coordination deficits that might have been induced by paraquat with or without poly(I:C) priming. The pole test was conducted once, during the second week after saline/poly(I:C) infusion. Latency to turn and latency to pole descent were both recorded for three trials. No behavioral difference were found for either of the measures across treatment groups (*Fs*_(5,42)_ < 1, *P* > 0.05; data not shown).

A mixed factor (time × treatment) ANOVA failed to reveal any significant difference in home-cage behavior in response to the paraquat and poly(I:C) treatments over time. Specifically, there was no significant time × treatment interaction nor was there an effect for time alone (*F*_(10,72)_ = 0.59; *F*_(2,72)_ = 1.77, respectively, *P* > 0.05). Similarly, the main effect for treatment did not reach significance (*F*_(5,72)_ = 2.07, *P* = 0.09). However, it was clear that there was a trend towards reduced home-cage activity; particularly at the second time point (that is, 22 days after the initial infusion) (Figure 1).

### **Quantification of tyrosine hydroxylase-positive neurons**

**Figure 2:**
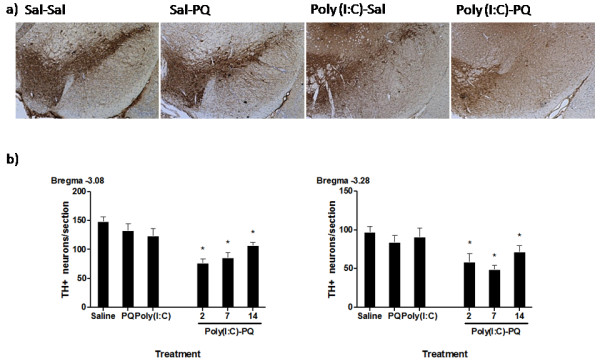
**Substania nigra pars compacta (SNc) tyrosine hydroxylase (TH) + neurons were quantified from multiple bregma levels.** Paraquat and poly(I:C) alone caused a modest, but non-significant TH + reduction relative to saline-treated controls. However, pre-treatment with poly(I:C) followed by paraquat induced a significant reduction in TH + neuronal counts at all bregma levels and at all re-exposure intervals. **(A)** Representative photomicrographs showing the degree of nigral TH + neuron loss in paraquat, poly(I:C), and the combination treatment groups compared with saline-treated controls. **(B)** Quantification of SNc TH + neuronal counts depicted in bar graphs. *P < 0.05, relative to saline-treated controls. All data expressed as mean ± SEM, n = 4 to 8.

Nigral DA cell bodies were counted at bregma levels −3.08, -3.16, 3.28, -3.40, and −3.52 for all treatment groups. Owing to unequal sample sizes across bregama levels stemming from a loss of some tissue sections at levels −3.16 and −3.52 (resulting in unbalanced analyses), a repeated-measures ANOVA was not conducted. However, separate ANOVAs for each level of the SNc did reveal that the number of nigral TH-positive neurons varied as a function of treatment at bregma levels −3.08, −3.28, −3.40 and −3.52 (F_5,29)_ = 8.67, *P* < 0.05; *F*_(5,30)_ = 4.654, *F*_(5,23)_ = 2.825, *F*_(5,24)_ = 3.724, *P* <0.05, respectively). However, significant variability at bregma level −3.16 precluded finding any significant difference at this particular SNc level (*F*_(5,21)_ = 1.560, *P* = 0.22). Groups treated with paraquat or poly(I:C) alone showed a modest, but non-significant decrease in SNc DA neurons (approx. 10 to 15%; Figure 2). However, poly(I:C) priming followed by subsequent paraquat administration led to a significant decrease in SNc DA neurons (25 to 50% relative to saline-treated controls; *P* < 0.05). Moreover, this effect was apparent when intervals of either 2, 7 or 14 days were interspersed between the poly(I:C) priming and subsequent initiation of the paraquat regimen.

**Figure 3:**
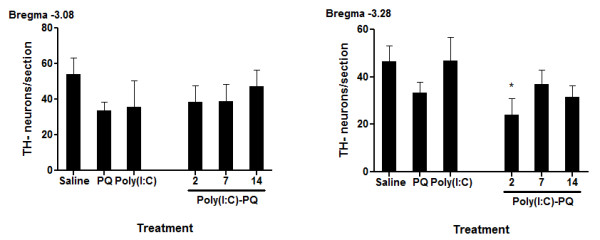
**Cresyl violet-stained tyrosine hydroxylase (TH)- neurons of the SNc from bregma level −3.08 to −3.40 were quantified as a measure of non-dopaminergic neurons.** Treatment with paraquat or poly(I:C) alone did not affect TH- neuron counts, but poly(I:C) pre-treatment followed 2 days later by paraquat administration induced a significant decrease in TH- neuron counts at bregma −3.28. *P < 0.05, relative to saline-treated controls. Data expressed as mean ± SEM, n = 4 to 7.

To ascertain whether the loss of TH-positive staining reflected a genuine neurodegenerative effect and whether the effect of paraquat was limited to DA neurons, all slides were counterstained with cresyl violet. Quantification of SNc cresyl violet-stained TH-negative neurons showed no significant differences between treatment groups (*F*_(5,28)_ = 0.804, *P* > 0.05, F_(5,29)_ = 1.791, *P* > 0.05; bregma levels −3.08 and −3.28, respectively; Figure 3). Hence, it appears that the effects of paraquat were more selective for TH-positive neurons, and that this was a genuine neurodegenerative effect. Indeed, increased cresyl violet TH-negative neuronal numbers would be expected if paraquat were simply inducing a suppression of TH expression in the absence of neuronal loss. However, if anything, paraquat provoked a trend towards reduced TH-negative neuronal counts that was reminiscent of that observed for the TH-positive DA neurons. Furthermore, combined exposure to poly(I:C) and paraquat caused a more marked effect. Specifically, *post hoc* comparisons showed that mice that received paraquat 2 days after poly(I:C) infusion displayed reduced TH-negative levels (at bregma level 3.28), relative to their saline-treated counterparts (*P* < 0.05). Thus, although the effects were much more pronounced for TH-positive SNc neurons, poly(I:C) infusion plus paraquat treatment appeared to affect non-DAergic neurons also.

### **Microglial assessment**

**Figure 4:**
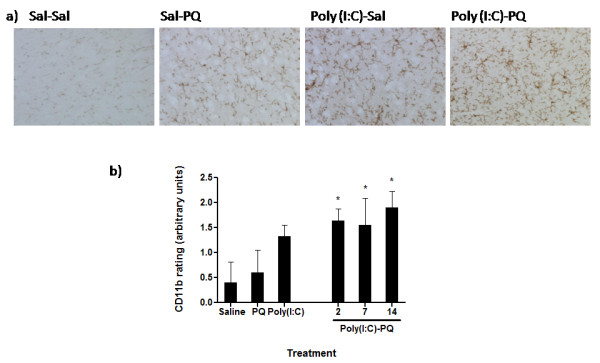
**Substania nigra pars compacta (SNc) sections were stained with anti-Cd11b and rated on morphological appearance as marker for microglial state.** Paraquat or poly(I:C) alone promoted a modest but non-significant elevation of microglial activation state relative to saline-treated controls. However, paralleling the TH + changes, poly(I:C) priming followed by later paraquat exposure significantly augmented signs of microglial activation. **(A)** Representative photomicrographs showing the degree of nigral microglial immunoreactivity induced by paraquat and poly(I:C) (note that the poly(I:C)-PQ image was taken from the group that received paraquat 2 days after poly(I:C) pre-treatment). **(B)** Quantification of ratings (arbitrary units) for microglial activation state. *P < 0.05, relative to saline-treated controls. Data expressed as mean ± SEM, n = 4 to 5.

Morphological changes in microglia were determined as a means of indicating differing degrees of glial activation, as a representation of the inflammatory state of the SNc. ANOVA showed that microglial ratings of morphological appearance approached significance as a function of the poly(I:C) and paraquat treatments (*F*_(5,19_) = 2.596, *P* = 0.05). Fisher’s *post hoc* comparison showed that although paraquat and poly(I:C) alone did not affect microglial ratings, enhanced ratings of glial morphology were evident in mice that were pre-treated with the viral analog and then later exposed to the pesticide (*P* < 0.05; Figure 4). However, as was the case for the TH-positive neuronal counts, the time interval between the poly(I:C) and paraquat treatments did not influence this outcome. In general, the poly(I:C) and paraquat combination provoked changes in morphology that were essentially characteristic of an intermediate microglial activation, with moderately compacted soma with shortened and thickened projections.

**Figure 5:**
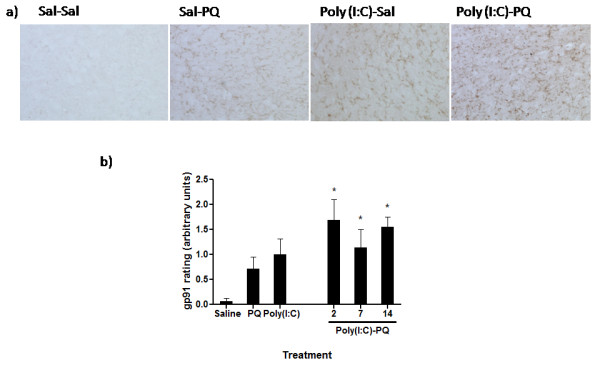
**Substania nigra pars compacta (SNc) sections were stained with anti-gp91**^**PHOX**^**as a marker for signs of oxidative stress.** In agreement with the CD11b ratings, poly(I:C) priming followed by later paraquat treatment resulted in significantly increased expression of gp91^PHOX^ immunostaining relative to saline-treated controls. **(A)** Representative photomicrographs showing the degree of gp91^PHOX^ immunoreactivity in paraquat, poly(I:C), and the combination treatment groups, (note that the poly(I:C)-PQ image was taken from the group that received paraquat 2 days after poly(I:C) pre-treatment), **(B)** Quantification of ratings (arbitrary scale units) of gp91^PHOX^ immunoreactivity in the SNc. * *P* < 0.05, relative to saline-treated controls. Data expressed as mean ± SEM, n = 5–8.

To assess the oxidative potential of activated microglia, SNc sections were stained with anti-gp91. This membrane-bound enzyme is responsible for the generation of the oxidative radical, superoxide. ANOVA showed that gp91 activity varied significantly between treatment groups (*F*_(5,23)_ = 3.166, *P* < 0.05). Administration of poly(I:C) or paraquat alone (Figure 5) induced a modest increase in gp91 activity (*P* = 0.06 and *P* = 0.11, respectively, relative to saline-treated mice). However, combined exposure of poly(I:C) and paraquat induced a very obvious elevation of gp91 immunoreactivity (*P* < 0.05), particularly in mice that received poly(I:C) 2 days before the paraquat injections, relative to saline treatment.

### **Striatal assessment**

Densitometry measures were used to quantify TH-positive terminals in the striatum. Unexpectedly, there were no differences found between groups with regard to striatal DA terminal coverage (*F* < 1; data not shown).

Given the lack of effect of paraquat upon striatal terminals and the paucity of behavioral changes, it was of interest to investigate the possibility that compensatory processes might have been upregulated over time in response to the pesticide exposure. To this end, striatal sections were immunostained for DCX (a measure of immature neurons), but we found that the total number of DCX-positive cells present in the subventricular zone (SVZ) ipsilateral to the poly(I:C) infusion did not significantly vary between the treatment groups (*F* < 1; data not shown). Similarly, new DCX-positive neurons did not seem to be induced to migrate from the SVZ into the striatum.

## **Discussion**

Accumulating evidence suggests that some combination of environmental toxins ranging from infectious insults to heavy metals and pesticides contribute to PD [21,31-35]. Some authors have even entertained the notion that a latent viral infection early in life may contribute to the onset of the disease [36]. Indeed, post-encephalitic cases of parkinsonism have been reported to occur years after viral infection [21,22]. It may be that exposure to immunological challenges (viral or bacterial) provoke modest neuroinflammation (for example, microglial activation, cytokine release) that over time may either: 1) cause frank neurodegeneration or 2) render DA neurons vulnerable to degeneration in response to subsequent environmental insults. With regard to this second possibility, we found that the double-stranded RNA viral analog poly(I:C) sensitized mice to the deleterious effects of paraquat exposure.

One of the most consistent findings across human post-mortem and animal toxin models of PD has been the association between strongly activated inflammatory microglia and DA neurodegeneration [8,37-41]. Emerging data also show that pro-inflammatory cytokines, particularly interferon (IFN)-γ and tumor necrosis factor (TNF)-α, modulate the microglial response to PD relevant environmental toxins [42-44]. In this study, we found that that poly(I:C) infusion alone caused modest but non-significant effects upon morphological status (using CD11b staining) and oxidative potential (assessed by increased gp91 staining) of microglia. This finding concurs with a previous reports indicating that a single poly(I:C) infusion (albeit of higher concentration) led to a decrease of TH-positive neurons in the SNc [25]. However, in the current study, the most dramatic effects were seen within the context of paraquat exposure, with poly(I:C) pre-treatment greatly increasing the degree of DA neuronal loss and microglial activation in response to later paraquat exposure.

In contrast to our previous findings using LPS priming [12], the present results indicated that timing between poly(I:C) priming and subsequent paraquat administration did not seem to be particularly important to the appearance of a sensitized response. In fact, poly(I:C) induced a protracted activation of microglia within the SNc that was still evident after 14 days, whereas in our previous study [12], we found that the effects of LPS upon CD11b-positive microglia were more transient (evident after 2 days, but returning towards baseline by 7 days). Although the effects of LPS and poly(I:C) were temporally distinct, the most important finding was that the augmented SNc DA neuronal loss was evident when exposure to the paraquat regimen occurred at a time of heightened microglial reactivity. However, it is noteworthy that the morphological changes of microglial (as indicated by CD11b staining) appeared to be most profound (although transient) with LPS priming (involving very compact amoeboid-like cells; [12]), compared with the more intermediate (but protracted) state induced by poly(I:C).

The differences in the temporal pattern of microglial immunoreactivity induced by poly(I:C) compared with previous reports using LPS might stem from variations in intracellular TLR-linked proteins. Indeed, inhibition of the TIRAP/MyD88 signaling proteins has been shown to block TLR4 but not TLR3 signaling [45]. Moreover, bone-marrow-derived macrophages treated with a TLR3 agonist displayed an enhanced and sustained immune response (that is, induction of type-1 IFN gene expression), relative to macrophages treated with a TLR4 agonist [45]. In addition, cultured human microglia were found to produce higher levels of some pro-inflammatory cytokines when treated with poly(I:C) compared with LPS [46].

Whatever the mechanism underlying TLR-linked pathways, oxidative stress factors are undoubtedly linked to PD-like pathology occurring after toxin exposure. Indeed, alterations in brain iron content, impaired mitochondrial function, and antioxidant protective systems, together with oxidative damage to lipids, proteins, and DNA, are typically seen in post-mortem brains of patients with PD and of toxin-exposed animals [47]. Post-mortem analysis of PD brains also showed upregulation of gp91 within the SNc [48,49]. Importantly, gp91 is the inducible catalytic subunit of the microglial NADPH oxidase enzyme, which in turn is responsible for the generation of the potentially damaging superoxide radical [50,51]. The fact that the most dramatic gp91 elevation seen in the present study occurred in poly(I:C) primed mice that also received paraquat suggests that the oxidative effect of paraquat is augmented in the context of an inflammatory microenvironment. This finding is consistent with the importance of gp91 shown in previous rodent studies that revealed attenuated degenerative effects of paraquat after genetic or pharmacological ablation of the NADPH oxidase subunit [48,52-54].

Although in our study, the most potent effects of paraquat were restricted to TH-positive SNc neurons, it should be mentioned that some TH-negative nigral neurons were also affected. These TH-negative cells probably represent a γ-Aminobutyric acid (GABA)ergic neuronal population [55]. This observation suggests that paraquat-induced neurodegeneration may not be entirely specific to DA neurons. It is possible that, given the marked pro-inflammatory and pro-oxidative state we observed in the SNc, non-DAergic neurons can succumb to such a volatile microenvironment. Indeed, LPS infusion alone was previously reported to decrease TH-negative neurons in the rat SNc [56], reinforcing the idea that strongly reactive microglia are sufficient to induce non-DAergic neuron death. It is conceivable that the transient disruption of the blood brain barrier expected after the surgical cannula implantation procedures could have augmented the overall entry of paraquat into the brain, and hence contributed to a non-specific loss of non-TH neurons.

Although paraquat has been reported to induce dose-dependent loss of SNc DA neurons [55], discrepant reports exist about the degree of degeneration in striatal terminals, with some studies reporting no effects of the pesticide [55,57]. In the present investigation, we found that both paraquat and poly(I:C), alone or in combination, did not affect DA terminals in the striatum. In agreement with this observation was the general lack of behavioral effects. In fact, there was only a modest transient but non-significant reduction of home-cage activity, which occurred 22 days after the poly(I:C) infusion. Furthermore, a pole test, which is designed to tap into forelimb and hindlimb deficits, failed to reveal any signs of coordination deficits.

Given the unexpected lack of striatal or behavioral deficits in the face of SNc soma loss, it was of interest to determine whether compensatory processes might have been provoked by poly(I:C) and paraquat. Indeed, the existence of neural stem cells and progenitors in the SVZ [58] raises the possibility that new neurons could be recruited into the nearby striatum after insult, as has been reported after ischemic brain injury [59-61]. Less evidence exists concerning the possibility of increased neurogenesis in a PD model; however, it has been reported that -methyl-4-phenyl-1,2,3,6-tetrahydropyridine (MPTP)-treated mice exhibited increased neurogenesis [62]. In the current study, both poly(I:C) and paraquat treatment failed to influence the number of DCX-positive immature neurons counted within the SVZ, and there was no evidence of new neuronal infiltration into the striatum. This negative finding might stem from the fact that, unlike another neurotoxin, MPTP, paraquat is not believed to be taken up by DA transporters at the terminals [63]. Alternatively, the magnitude of the SNc soma lesion might simply have been insufficient to affect processes at the downstream terminals.

## **Conclusions**

Overall, the present findings emphasize the importance of inflammatory and oxidative factors and reinforce the idea that PD might be triggered by cumulated exposures to multiple environmental insults (possibly in combination with genetic vulnerabilities). These data indicate that viral infection might ‘set the stage’ or essentially sensitize neuroinflammatory responses in such as way that subsequent environmental insults induce exaggerated effects. Future therapies for PD might consider targeting crucial inflammatory mechanisms that are often triggered by environmental exposures to various immune or chemical toxins.

## **Abbreviations**

PD, Parkinson’s disease; SNc, Substantia nigra pars compacta; ROS, Reactive oxygen species; MPTP, 1-methyl-4-phenyl-1,2,3,6-tetrahyropyridine; NADPH, Nicotinamide adenine dinucleotide phosphate; BDNF, Brain-derived neurotrophic factor; LPS, Lipopolysaccharide; TNF, Tumor necrosis factor; IFN, Interferon; Bcl-2, B-cell lymphoma 2; TLR, Toll-like receptor; PAMP, Pathogen-associated molecular pattern; MyD88, Myeloid differentiation factor-88; NF-κB, Nuclear factor κB; poly(I:C), Polyinosinic:polycytidylic acid poly; TH, Tyrosine hydroxylase; DCX, Doublecortin; DAB, Diaminobenzidine; SVZ, Subventricular zone; GABA, Gamma-Aminobutyric acid; TIRAP, TIR homology domain-containing adaptor protein.

## **Competing interests**

The authors declare that they have no competing interests.

## **Authors’ contributions**

JB carried out microglial immunohistochemistry studies and wrote the first draft of the manuscript, ENM quantified dopamine neuronal loss and performed surgery and central infusions, AG, EN, KM and MC helped with animal injections and behavioral testing, SH designed the studies, interpreted the data and wrote the final version of the manuscript. All authors read and approved the final manuscript.
